# Development and characterization of a recombinant Senecavirus A expressing enhanced green fluorescent protein

**DOI:** 10.3389/fmicb.2024.1443696

**Published:** 2024-09-26

**Authors:** Weihong Huang, Yongjie Chen, Ting Xu, Ting Xiong, Yadi Lv, Dingxiang Liu, Ruiai Chen

**Affiliations:** ^1^College of Veterinary Medicine, South China Agricultural University, Guangzhou, China; ^2^Zhaoqing Branch of Guangdong Laboratory of Lingnan Modern Agricultural Science and Technology, Zhaoqing, China; ^3^Integrative Microbiology Research Centre, South China Agricultural University, Guangzhou, China

**Keywords:** Senecavirus A, infectious clone, eGFP reporter virus, mouse infection, neutralizing antibody

## Abstract

**Introduction:**

Senecavirus A (SVA), belonging to the genus *Senecavirus* in the family *Picornaviridae*, is an emerging pathogen causing vesicular disease in pigs. The main clinical manifestations of SVA infection include high mortality in neonatal piglets, skin ulceration, and vesicular lesions. So far, there is no commercially available vaccines or drugs against SVA. Construction of SVA infectious clones carrying reporter genes will help understand the characteristics of SVA and promote vaccine development.

**Methods:**

In this study, we established a reverse genetics system for a local SVA isolate and used it to rescue a recombinant SVA, rSVA-eGFP, expressing the enhanced green fluorescent protein (eGFP) by inserting eGFP, GSG linker and the P2A sequence between 2A and 2B genes.

**Results:**

We found that rSVA-eGFP exhibited a high replication efficiency comparable to the parental virus, was able to express the eGFP reporter efficiently and stable in maintaining the reporter gene up to six rounds of serial passages in BHK-21 cells. In mice, rSVA-eGFP also showed similar replication kinetics and pathogenicity to the parental virus, both causing mild lung lesions. In addition, a high-throughput viral neutralization assay was developed using eGFP as a surrogate readout in a fluorescence-based direct titration (FBT) assay based on rSVA-eGFP, facilitating rapid and accurate determination of the neutralizing antibody (nAb) titers.

**Discussion:**

The successful establishment of an SVA reverse genetics system and the rescue of rSVA-eGFP would create a powerful tool for future studies of SVA replication mechanisms and pathogenicity as well as for antiviral development.

## 1 Introduction

Senecavirus A (SVA), also known as Seneca Valley virus (SVV), belongs to the genus *Senecavirus* in the family *Picornaviridae*, and is the sole member of the genus *Senecavirus* with only one serotype (Hales et al., 2008; Zhang et al., 2018). SVA (SVV-001) was first identified as a cell culture contaminant (Reddy et al., 2007). Between 1988 and 2005, six picorna-like isolates from pigs in the United States were found to be genetically and serologically related to SVA (Zhang et al., 2018). In 2007, the first SVA-positive case was reported in Canada (Pasma et al., 2008). Since 2014, SVA infection has been reported in several countries without previous outbreaks of SVA infection, including Brazil, Colombia, Thailand, Vietnam, and Chile (Vannucci et al., 2015; Sun et al., 2017; Saeng-chuto et al., 2018; Arzt et al., 2019; Bennett et al., 2022). In China, SVA was first reported and isolated in Guangdong province in 2015, and subsequently nationwide (Qian et al., 2016; Wang et al., 2017; Wu et al., 2017; Zhu et al., 2017; Peng et al., 2020; Wang H. et al., 2020). Representative clinical symptoms of SVA-infected pigs include high mortality in neonatal piglets, skin ulceration and vesicular lesions, indistinguishable from vesicular diseases caused by foot-and-mouth disease virus, swine vesicular disease virus, vesicular stomatitis virus and vesicular exanthema of swine virus (Vannucci et al., 2015; Zhang et al., 2018). Furthermore, reports of asymptomatic SVA-infected cases are increasing, and in addition to pigs, cattle have also been reported to serve as natural hosts of SVA, which increases the difficulty in SVA prevention and control (Zhang Z. et al., 2019; Zhou et al., 2021).

SVA is a single-stranded, positive-sense RNA virus. The RNA genome is approximately 7,300 nucleotides in length, and contains a single open reading frame (ORF) encoding a polyprotein of 2,181 amino acids, flanked by a 5′ untranslated region (UTR) and a 3′UTR with a polyadenosine (polyA) tail. The polyprotein undergoes a series of proteolytic processes to produce structural proteins VP4, VP3, VP2, and VP1, and nonstructural proteins L, 2A, 2B, 2C, 3A, 3B, 3C, and 3D (Hales et al., 2008; Wang et al., 2023).

Construction of recombinant SVA reporter viruses would facilitate the virological research and antiviral development. Current strategy used to construct picornaviruses as vectors to express foreign genes exploited the ribosomal skipping function of the 2A sequence. The insertion position of foreign genes is generally either between 5′UTR and ORF as an N-terminal addition of the viral polyprotein, or in the upstream or downstream of the viral 2A gene as a 2A-foreign gene-2A cassette (Seago et al., 2013; Xu et al., 2015; Han et al., 2018; Guo et al., 2023). One application of recombinant reporter viruses was to use the expression of the reporter gene as a surrogate readout for viral infection in virus neutralization assay (Chen et al., 2016; Liu et al., 2020a; Guo et al., 2021). However, this approach appears to share the same shortcomings as the standard virus neutralization assay that uses cytopathic effect (CPE) as a readout, which is cumbersome and not high-throughput.

In this study, eGFP was selected as the reporter gene, combined with the P2A sequence, to construct infectious clones from a local SVA isolate. The insertion of the reporter gene and elements between the 2A and 2B genes was selected as the optimal method for the rescue of rSVA-eGFP, with the introduction of an additional optimal GSG linker to improve the skipping efficiency of 2A. The recombinant virus shares similar growth properties and pathogenicity to the parental virus *in vitro* and in mice, and is genetically stable for at least six passages with consistent high-level expression of the eGFP reporter gene. Furthermore, a high-throughput FBT assay was developed based on this recombinant reporter virus for the detection of nAb. By measuring fluorescence intensity, the application of rSVA-eGFP simplifies traditional nAb detection using CPE as a readout. This recombinant reporter virus would become a useful system for studies of the replication mechanisms and pathogenicity of SVA as well as for antiviral development, with a promising application potential.

## 2 Materials and methods

### 2.1 Cell, virus, and antibody

Baby hamster kidney-21 (BHK-21) cells and mouse embryonic hepatocytes (BNL-CL.2) cells were maintained in a humidified atmosphere incubator of 5% CO_2_ at 37°C in Dulbecco’s modified Eagle’s medium (DMEM) supplemented with 1% penicillin/streptomycin and 10% fetal bovine serum (FBS). The parental SVA strain (Genbank access No.: PP974472) was isolated from porcine lymph node and was propagated in BHK-21 cells. Anti-β-actin mouse monoclonal antibody (Cat. No. HC201) and anti-GFP mouse monoclonal antibody (Cat. No. HT801) were obtained from TransGen Biotech (Beijing, China). Mouse monoclonal anti-dsRNA antibody (J2) (Cat. No. 76651) was obtained from Cell Signaling Technology (Danvers, MA, United States). Alexa Fluor 555-labeled Donkey Anti-Mouse IgG (H + L) (Cat. No. A0460), Alexa Fluor 555-labeled Donkey Anti-Rabbit IgG (H + L) (Cat. No. A0453) and Alexa Fluor 488-labeled Goat Anti-Mouse IgG (H + L) (Cat. No. A0428) secondary antibodies were obtained from Beyotime (Shanghai, China). IRDye 800CW goat anti-mouse IgG (H + L) (926–32,210) and IRDye 800CW goat anti-rabbit IgG (H + L) (926–32,211) were obtained from Li-COR Biosciences (Lincoln, NE, United States).

### 2.2 Generation of recombinant viruses

Viral RNA was extracted from the passage 3 virus from BHK-21 cells using TRIzol (Invitrogen), and reversed transcribed with PrimeScript^™^ II 1st Strand cDNA Synthesis Kit (Takara, Japan). Using the viral cDNA as the template, viral fragment A, B and C were constructed through RT-PCR with overlapping primers (Supplementary Table S1). The 5′ and 3′ ends of the genome were further determined using HiScript-TS 5′/3′ RACE Kit (Vazyme, China), followed by TA cloning and sequencing. Viral fragment D and E, that covering the 5′UTR and 3′UTR, were synthesized by Synbio Technologies (Suzhou, China). The five fragments were cloned into linearized pBR322 vector via homologous recombination with ClonExpress II One Step Cloning Kit (Vazyme, China). The cytomegalovirus (CMV) enhancer, CMV promoter and hammerhead ribozyme (HamRbz) element were sequentially inserted upstream of the 5′UTR, while hepatitis D virus (HDV) ribozyme and bGH poly(A) signal element were following the 3′UTR. To differentiate the infectious clone derived virus from the parental clinical isolate, a MfeI restriction endonuclease site was introduced with a C6761A synonymous nucleotide mutation as a molecular marker. The resulting plasmid containing the full-length SVA genome was designated as pBR322-SVA. In addition, fragment C was cloned into linearized pBR322 vector to generate standard plasmid pBR322-P3, used for viral RNA quantification.

Based on this infectious clone, five infectious cloning schemes of SVA-eGFP were developed. In scheme 1, the eGFP gene (Genbank access No.: U55761) without a stop codon at its 3′ end and porcine teschovirus-1 2A (P2A) element (Supplementary Table S2) were inserted between the 2A gene and 2B gene in pBR322-SVA, and the resulting plasmid was designated as pSVA-eGFP-1. In scheme 2, pSVA-eGFP-2 was constructed by introducing an addition GSG linker (Supplementary Table S2) between the eGFP gene and P2A element in pSVA-eGFP-1. In scheme 3 and 4, GSG linker in pSVA-eGFP-2 was replaced with SSG or GGG linker (Supplementary Table S2), respectively, generating pSVA-eGFP-3 with SSG linker and pSVA-eGFP-4 with GGG linker. In scheme 5, the eGFP gene, GSG linker and P2A element were inserted between 5′UTR and L gene in pBR322-SVA, generating plasmid pSVA-eGFP-5. Screening was performed via Sanger sequencing to confirm the introduced mutation as well as genetic integrity of the rest of the fragment.

Plasmid DNA (2 μg/well) was transfected into BHK-21 cells on a 6-well plate using Lipofectamine 2000 (Thermo Fisher, Waltham, MA, United States). After incubation for 12 h, medium was replaced with Opti-MEM (Gibco) to continue culturing. Cells were harvested at 48 h post-transfection, lysed by frozen and thawed three times, the supernatant was collected by centrifugation, and blind-passaged on BHK-21 cells for three more passages.

### 2.3 Indirect immunofluorescence assay

Cells were seeded overnight and infected with virus at a multiplicity of infection (MOI) of 0.1. At 12 h post-infection, cells were fixed with 4% paraformaldehyde for 30 min, permeabilized with 0.3% Triton X-100 for 10 min, and blocked with 5% bovine serum albumin (BSA) at 37°C for 1 h. Cells were then incubated with primary antibodies at 4°C overnight, washed three times with PBS, and stained with secondary antibodies at 37°C for 1 h in the dark, followed by washing three times. Finally, cells were stained with DAPI and analyzed by an EVOS M5000 fluorescence imaging system (Thermo Scientific, United States).

### 2.4 Western blotting

Cells were lysed with NP-40 lysis buffer (Beyotime, China) containing the protease inhibitor phenylmethylsulfonyl fluoride (PMSF). Lysates were centrifuged, and 120 μL of the resulting supernatants were mixed with 30 μL of 5× SDS-PAGE sample loading buffer (Beyotime, China). Protein samples were heated at 98°C for 15 min and centrifuged at 12,000 rpm for 1 min, and the supernatants were loaded to 10% SurePAGE (GenScript, China). Sodium dodecyl sulfate-polyacrylamide gel electrophoresis (SDS-PAGE) was performed with the Bio-Rad Mini-PROTEAN Tetra cell system, and proteins were further electrophoretically transferred to nitrocellulose membranes using the Bio-Rad TransBlot protein transfer system. The membrane was blocked with 5% skim milk for 1 h, and incubated with the primary antibody overnight at 4°C. After washing three times with Tris-buffered saline with Tween 20 (TBST), the membrane was incubated with the corresponding secondary antibody for 1 h at room temperature in the dark. The membrane was washed three times, and protein bands were visualized using an Azure c600 imager (Azure Biosystems, United States).

### 2.5 Analysis of the stability of the recombinant virus

The rescued rSVA-eGFP was continuously passaged on BHK-21 cells for 11 passages. Each passage was harvested, inoculated on BHK-21 cells in a 6-well plate, and the fluorescence intensity was examined at 12 h post-infection by fluorescent microscopy. To analyze the stability of the exogenous gene eGFP in rSVA-eGFP, RT-PCR analysis was performed with the eGFP primer pair (Supplementary Table S1). The amplified products were analyzed by agarose gel electrophoresis, purified using Cycle-Pure Kit (Omega, United States) and sequenced.

### 2.6 Growth kinetics

The replication kinetics of wtSVA, rSVA and rSVA-eGFP were characterized in BHK-21 cells infected with each of these viruses at a MOI of 0.1. After incubation for 1 h, cells were washed once with PBS and incubated with DMEM containing 2% FBS at 37°C. Supernatants were collected at 6, 12, 24, 36, 48, and 72 h post-infection, respectively, and subjected to 50% tissue culture infective dose (TCID_50_) assay. Briefly, 100 μL of 10-fold serially diluted sample was added to monolayers of BHK-21 cells on 96-well plates, with 4 replicate wells for each dilution. After incubation for 48 h, the wells were determined to be either positive (with CPE) or negative (without CPE), and the TCID_50_ was calculated based on the Reed-Muench method (Yamada and Liu, 2009). Data are expressed as the mean ± SD of three independent experiments.

### 2.7 Plaque assay

One hundred and ten microliter of virus was mixed with 990 μL of DMEM to perform 10-fold serial dilutions, and 900 μL of the diluted viral preparation was inoculated into BHK-21 cells in a 6-well plate. After incubation for 1 h, cells were washed once with PBS, and incubated with 2 mL of DMEM containing 2% FBS and 1% low melting point agarose at 37°C for 3 days. Cells were then fixed with 4% paraformaldehyde and stained with 1% crystal violet.

### 2.8 rSVA-eGFP-based neutralization assay

Antiserum was diluted 1:10 after heat-inactivation at 56°C for 30 min, followed by 4-fold serial dilution (1:40 to 1:655360) in DMEM containing 2% FBS, and then incubated with an equal volume of P5 rSVA-eGFP containing 200 TCID_50_ at 37°C for 1 h. The mixtures were added to a monolayer of BHK-21 cells on 96-well plate, with 4 replicate wells for each dilution. After incubation for 48 h, eGFP signals were determined using an EVOS M5000 fluorescence imaging system and Varioskan LUX multimode microplate reader (Thermo Scientific, United States).

Each experiment was set up with mock (uninfected cells), positive control (inoculation with the mixture of positive serum and rSVA-eGFP), and negative control (inoculation with the mixture of negative serum and rSVA-eGFP). Green fluorescent signals should be observed in the negative control, but not in the mock and positive control. In addition, to verify the accuracy of virus dilution, the rSVA-eGFP was diluted as 0.1, 1, 10, and 100 TCID_50_ and infected BHK-21 cells on 96-well plate, with 8 replicate wells for each dilution. Green fluorescent signals should be positive in all wells of 100 and 10 TCID_50_ infection group, approximately half of the wells were positive in 1 TCID_50_ infection group, and all wells were negative in 0.1 TCID_50_ infection group. The nAb titer was calculated using Reed-Muench method, representing the serum dilution that renders 50% reduction in the number of wells with CPE or fluorescent signals.

### 2.9 Mouse infection experiment

Sixty 3-week-old female C57BL/6 J mice (Charles River, Beijing, China) were randomly divided into wtSVA infection (*n* = 20), rSVA-eGFP infection (*n* = 20) and PBS control (*n* = 20) groups. After isoflurane anesthesia, mice of the infection groups were intranasally inoculated with 50 μL of wtSVA or P5 rSVA-eGFP (10^8^ TCID_50_), and the control group the same volume of PBS. Clinical symptoms and body weight were recorded daily for 14 days. At 1, 3, 5, 7, and 14 days post-infection (dpi), three mice in each group were euthanized, and lungs, liver, spleen, brain, heart, and kidneys were collected, homogenized in 200 mg/mL (weight/volume) DMEM, and centrifuged. Viral RNA was extracted from the supernatants using RNA Rapid Extraction Kit (Fastagen, China; no. 220011), and reverse-transcribed with HiScript III All-in-one RT SuperMix (Vazyme, China). The cDNA was then used as a template for quantitative PCR (qPCR) with the ChamQ Universal SYBR qPCR Master Mix (Vazyme, China) and the gene specific primers listed in Supplementary Table S1. In addition, a standard curve was generated with pBR322-P3 and used to quantify the total amount of viral RNA in each sample. Threshold cycle (C_T_) values for virus samples were plotted against the standard curve to obtain viral copy numbers. All RT-qPCR experiments were performed using a CFX96 real-time system (Bio-Rad, United States).

For histology assay, collected tissues at 3 dpi including lungs, liver, spleen, brain, heart, and kidneys were fixed in 4% PFA (pH 7.4) and embedded in paraffin. Hematoxylin and eosin (H&E) staining was conducted for lesion evaluation.

### 2.10 Statistical analysis

All the viral RNA copies and titration data were obtained from at least three repeated experiments. The data were expressed as means ± standard deviations. Two-way analysis of variance was used for statistical analysis in GraphPad Prism 8.0 software (GraphPad, United States). *p* < 0.05 was considered significant (*p < 0.05, **p < 0.01). Correlation analysis was performed using Pearson correlation with 95% confidence interval. In determining the standard curve of FBT assay, second-order polynomial was used for nonlinear fitting of relative fluorescence unit (RFU) and nAb titer.

## 3 Results

### 3.1 Construction of an infectious clone from a SVA local isolate

**Figure 1:**
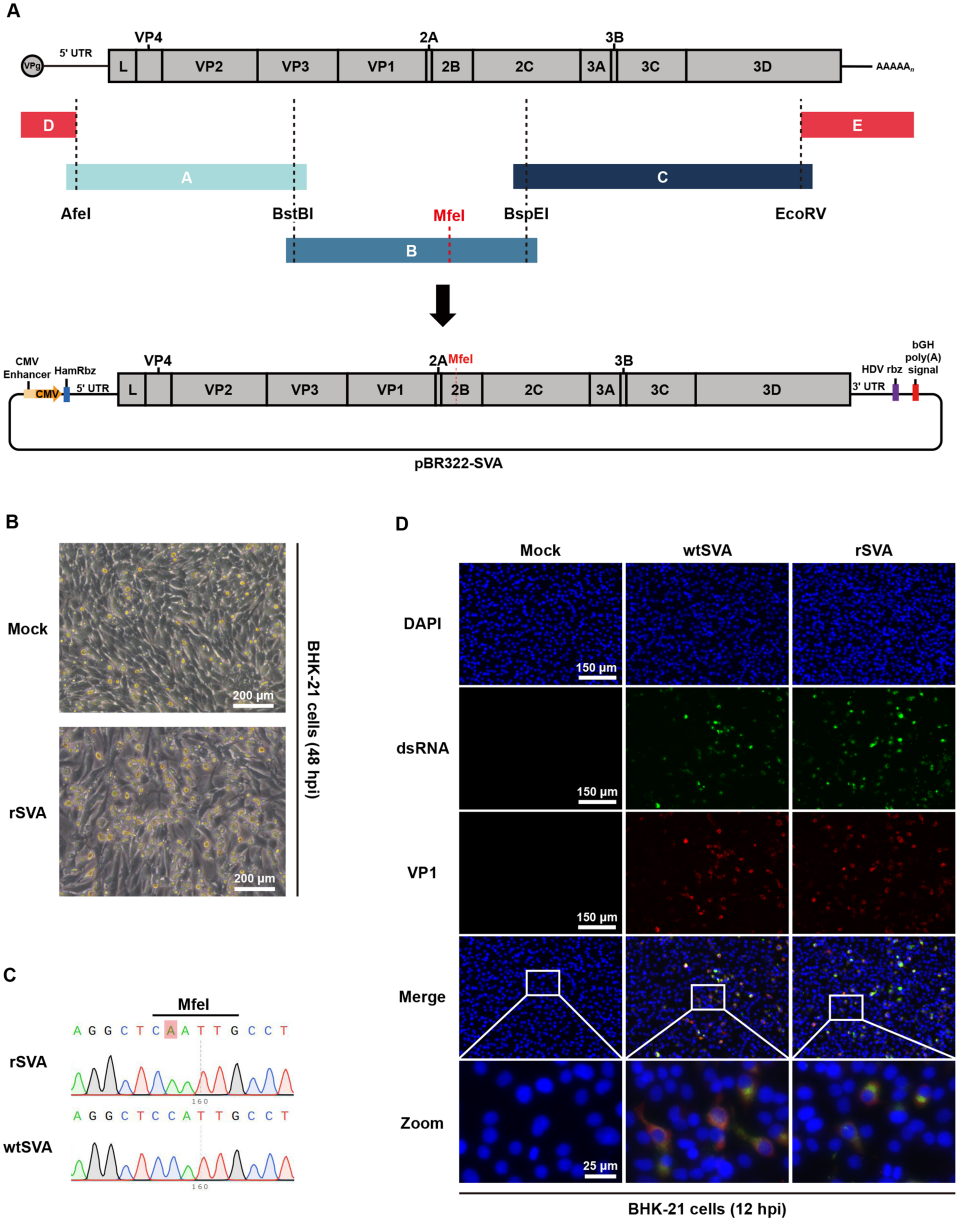
Construction and characterization of an SVA infectious clone. **(A)** Diagram of SVA genome structure and construction strategy for the full-length infectious clone of SVA. **(B)** SVA infectious clone was transfected into BHK-21 cells, and cytopathic effect (CPE) was observed 48 h post-transfection. **(C)** Chromatograms of nucleotide sequencing results, and the engineered nucleotide mutation as a molecular maker for the recombinant virus was indicated. **(D)** BHK-21 cells were mock treated or infected with wtSVA and rSVA (MOI = 0.1), respectively, and immunostained with anti-dsRNA antibody and anti-VP1 antibody at 12 hpi. Cell nuclei were stained with DAPI.

As illustrated schematically in Figure 1A, RT-PCR was performed using viral RNA as a template, and three amplified fragments and two synthetic fragments were cloned into a linearized pBR322 vector. To differentiate the infectious clone-derived virus from the parental virus, a C6761A synonymous mutation was introduced as a genetic marker. The full-length SVA clone, pBR322-SVA, was transfected into BHK-21 cells, and typical CPE was observed at 48 h post-transfection (Figure 1B). Whole-genome sequencing of the rescued rSVA showed that the recombinant virus retained the C6761A synonymous nucleotide mutation without additional sequence changes, excluding the potential contamination from the parental virus (Figure 1C). More importantly, double-stranded RNA (dsRNA) and viral VP1 protein were detected in infected BHK-21 cells by immunofluorescence assay. Both wtSVA- and rSVA-infected cells showed punctate staining of dsRNA, indicating the formation of viral genome replication intermediates (Figure 1D). The dsRNA fluorescence signals were shown to overlap with the VP1 protein fluorescence signals, while no signal was observed in uninfected cells (Figure 1D). These results all demonstrated the successful rescue of a recombinant SVA and the establishment of an infectious clone system for this local SVA isolate.

### 3.2 Construction and optimization of cloning strategies for the rescue of recombinant SVA expressing eGFP

**Figure 2:**
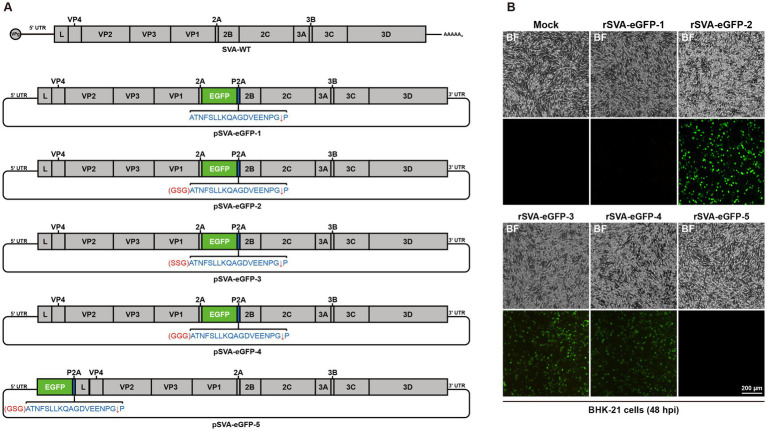
Generation of eGFP-expressing SVA. **(A)** Construction strategies for the full-length infectious clones of eGFP-expressing SVA. **(B)** Different infectious clones of eGFP-expressing SVA were transfected into BHK-21 cells, and CPEs and eGFP fluorescence signals were observed at 48 h post-transfection.

Based on the full-length pBR322-SVA, eGFP-expressing SVA clones were generated in five construction strategies (Figure 2A; Supplementary Table S3). The pSVA-eGFP-1 was constructed by inserting the eGFP gene and the P2A element between 2A and 2B genes in the genome, and the expression of separate eGFP was achieved by the ribosomal skipping mechanism of the 2A polypeptide. Previous studies have used this method to rescue a reporter SVA virus (Chen et al., 2016; Li et al., 2019; Liu et al., 2020a). However, in this study, no fluorescence signal and CPE were observed after transfection of pSVA-eGFP-1 (Figure 2B), demonstrating that this construction strategy failed to successfully rescue rSVA-eGFP-1. It was speculated that the skipping efficiency of P2A might be affected by the inserted eGFP reporter gene, because the 2A reaction may be inhibited by the C-terminal regions of proteins immediately upstream of P2A when translocated to the endoplasmic reticulum (De Felipe et al., 2010). Introduction of a GSG linker immediately upstream of P2A may improve the skipping efficiency of P2A (Chng et al., 2015; Park et al., 2016). Therefore, the GSG linker was inserted between the eGFP gene and the P2A element to construct pSVA-eGFP-2 (Figure 2A), and eGFP fluorescence signals and CPEs were observed after transfection (Figure 2B), confirming the rescue of rSVA-eGFP-2 after optimization of the construction strategy.

Since the reporter SVA was successfully rescued by the introduction of a GSG linker, to further explore whether amino acid changes in the linker would affect virus rescue, the GSG linker was replaced with SSG (pSVA-eGFP-3) and GGG (pSVA-eGFP-4), respectively (Figure 2A). Transfection of these two full-length clones into BHK-21 cells caused CPEs and the detection of slightly weaker eGFP fluorescence signals than did rSVA-eGFP-2 (Figure 2B). The rescue of rSVA-eGFP-3 and rSVA-eGFP-4 demonstrated the essential requirement for the inclusion of linker sequences in the infectious clones, but the exact amino acid sequence would also affect the eGFP fluorescence expression.

The genomic position of SVA suitable for the insertion of a reporter gene was then characterized. As for Enterovirus 71 (EV71) and Coxsackievirus A16 (CA16), which also belong to the *Picornaviridae* family, reporter viruses were rescued from infectious clones containing reporter genes between 5′UTR and VP4 gene (Caine and Osorio, 2017; Yu et al., 2023). This strategy was attempted to rescue a reporter SVA, by introducing the eGFP gene, GSG linker and P2A element between 5′UTR and L gene in pSVA-eGFP-5. Neither fluorescence signal nor CPE was observed after transfection of this construct (Figures 2A,B), indicating that this site was not suitable for the insertion of foreign genes.

Taken together, these results demonstrated that choosing an appropriate insertion site for foreign genes and introducing a linker are essential for successful rescue of the reporter SVA. Comparing the CPEs and eGFP fluorescence expression, the reporter virus derived from the infectious clone containing a GSG linker had a better performance than the other reporter viruses. This recombinant virus derived from pSVA-eGFP-2, was designated as rSVA-eGFP and used in subsequent studies.

### 3.3 Characterization of the growth kinetics of rSVA-eGFP in culture cells

**Figure 3:**
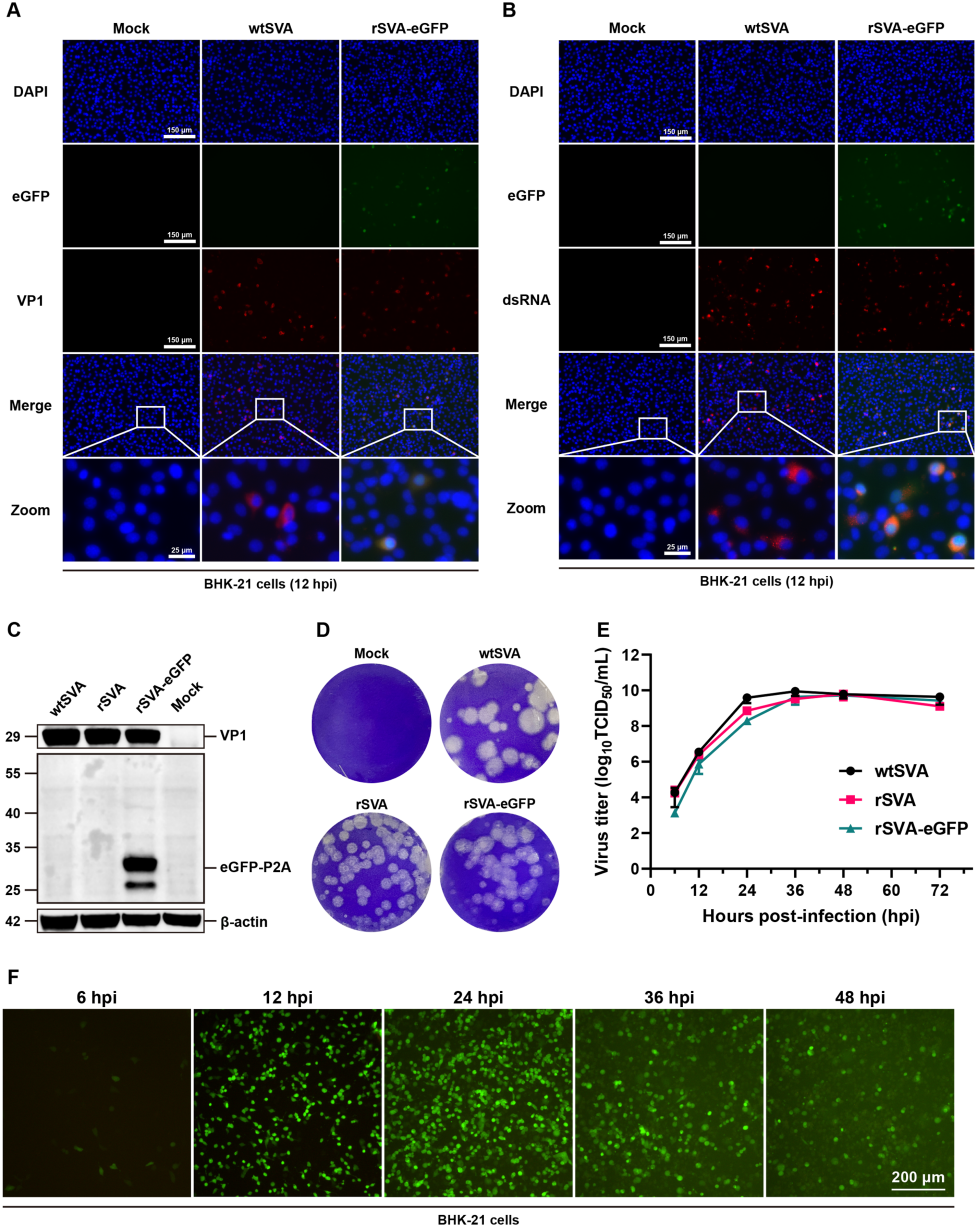
Characterization of rSVA-eGFP in culture cells. BHK-21 cells were mock-treated or infected with P5 rSVA-eGFP (MOI = 0.1), and immunostained with anti-VP1 antibody **(A)**, and anti-dsRNA antibody **(B)** at 12 hpi. Cell nuclei were stained with DAPI. **(C)** BHK-21 cells were mock-treated or infected as in **(A)**, and immunoblotted with anti-VP1 and anti-GFP antibodies, respectively. β-actin was used as a loading control. **(D)** Plaque morphology of wtSVA, rSVA and P5 rSVA-eGFP in BHK-21 cells. 110 μL of the initial virus stock was used for 10-fold serial dilutions, and 900 μL of the diluted samples were used to infect BHK-21 cells. Cells either infected with an indicated virus at a dilution of 10^−6^ or mock-treated were stained with 1% crystal violet at 72 hpi. **(E)** Growth curve of wtSVA, rSVA and P5 rSVA-eGFP in BHK-21 cells (MOI = 0.1). Cell supernatants were collected at 6, 12, 24, 36, 48, and 72 hpi for virus titration by TCID_50_ assay. **(F)** The live fluorescence-expressing BHK-21 cells were observed in cells infected with P5 rSVA-eGFP (MOI = 1) at 6, 12, 24, 36, and 48 hpi.

The growth kinetics and properties of rSVA-eGFP in culture cells were then characterized. Using Western blotting and immunofluorescence staining, we detected the expression of viral VP1 protein and viral RNA replication intermediates in BHK-21 cells infected with wtSVA, rSVA and rSVA-eGFP, respectively. Overlapped fluorescence signals of VP1 protein and dsRNA with eGFP were detected in rSVA-eGFP-infected cells (Figures 3A,B). The eGFP-P2A fusion protein with an apparent molecular mass of 29 kDa was also detected in rSVA-eGFP-infected cells, a putative 44 kDa band, representing the eGFP-P2A-2B fusion protein caused by a potential skipping failure, was not detected (Figure 3C). These results demonstrate the high efficiency of the P2A skipping activity in this construct.

Additionally, plaque morphologies and viral growth curves were compared among wtSVA, rSVA and rSVA-eGFP. As shown in Figure 3D, plaques formed in cells infected with rSVA and rSVA-eGFP were slightly smaller than those in cells infected with wtSVA. Consistent with the results of Western blotting and immunofluorescence staining, rSVA-eGFP could replicate efficiently, although with slightly lower than wtSVA at 12–24 hpi (Figure 3E). Examination of fluorescence-expressing cells in time-course experiments showed that positive cells were first detected as early as at 6 hpi, increased rapidly at 12–24 hpi, but decreased at 36–48 hpi, likely due to the massive death of the infected cells (Figure 3F). Overall, the data showed that rSVA-eGFP is replication competent and infectious, although the viral replication efficiency is slightly lower than that of wtSVA.

### 3.4 Characterization of the genetic stability of rSVA-eGFP in culture cells

**Figure 4:**
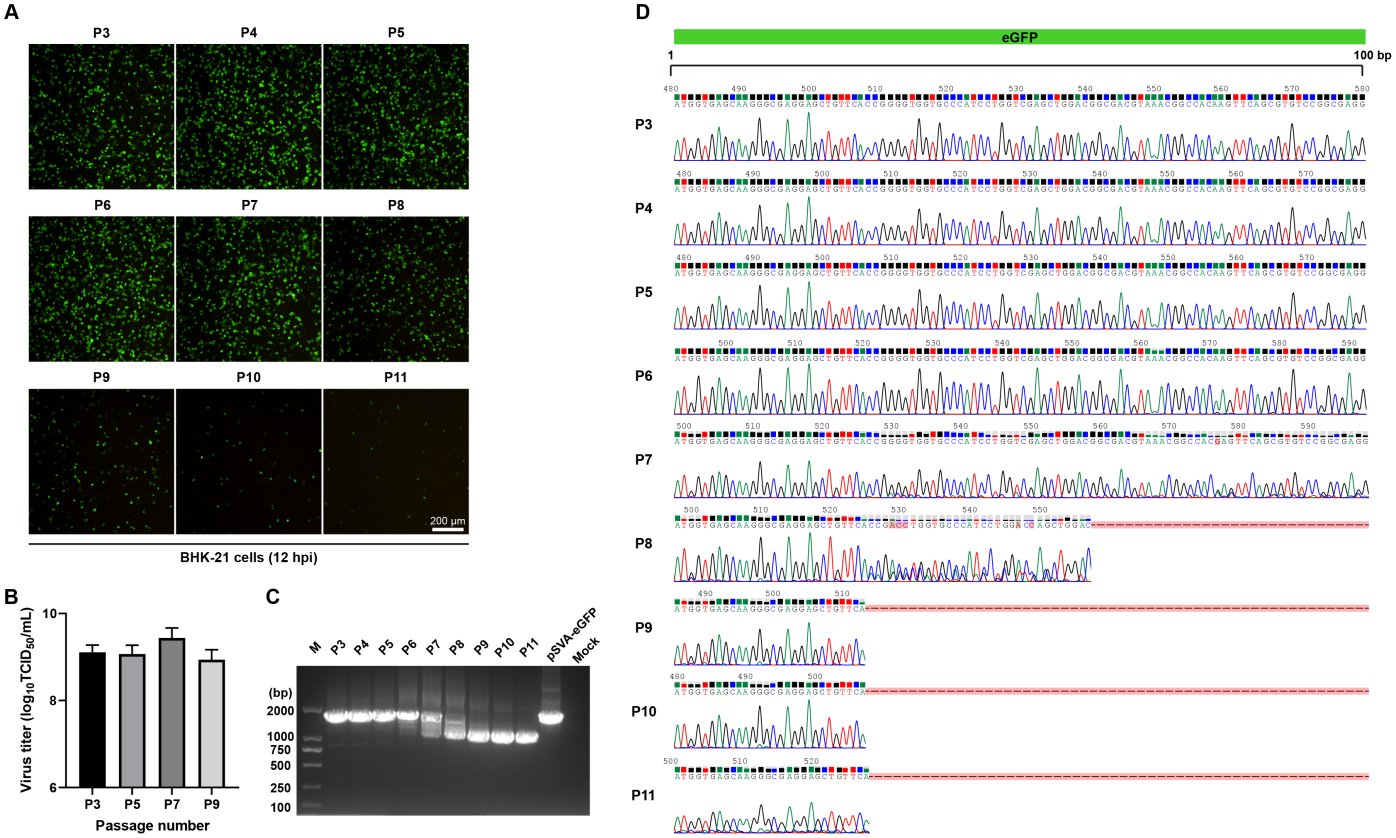
The genetic stability of rSVA-eGFP reporter virus. **(A)** The expression of eGFP in BHK-21 cells infected with different passages of rSVA-eGFP (MOI = 1) at 12 hpi. **(B)** Virus titration of each passage of rSVA-eGFP. The P3, P5, P7 and P9 recombinant reporter viruses were subjected to TCID_50_ assay. **(C)** RT-PCR analysis of different passages of rSVA-eGFP. BHK-21 cells were infected with P3-P11, and RT-PCR analysis was performed to verify the integrity of eGFP gene. **(D)** Sequencing chromatograms of regions covering the eGFP gene fragment (0–100 bp). The RT-PCR amplified fragments presented in **(C)** were sequenced and the sequence alignment analysis was performed based on the eGFP gene sequence. The mutations or deletions were indicated.

From the Western blotting data presented in Figure 3C, a smaller band of about 25 kDa was also detected with anti-GFP antibodies, in addition to the 29 kDa eGFP-P2A fusion protein. The reason for the detection of this product is not certain, but it may be caused by viral protease-mediated, partial cleavage of eGFP. Alternatively, it may be due to the instability of the recombinant virus, resulting in partial deletion of eGFP from the recombinant virus. This prompted further studies of the stability of eGFP gene in rSVA-eGFP by serial passages in BHK-21 cells, revealing that eGFP fluorescence signals were relatively constant at P3-P7, but were gradually decreased from P8 (Figure 4A). As the titers of rSVA-eGFP remained relatively constant during serial passages (Figure 4B), the integrity of the eGFP gene in rSVA-eGFP was determined using RT-PCR. It confirmed that the eGFP gene with the expected size was detectable at P3-P7, but the amplified fragments were smaller after P8 (Figure 4C). Nucleotide sequencing verified that there were no overlapping peaks and no change in the eGFP sequence from P3 to P6 (Figure 4D). Overlapping peaks were detected from P7 to P11, with mutations or deletions in the eGFP sequence (Figure 4D). These results confirmed that rSVA-eGFP could stably retain the eGFP reporter gene within the first six rounds of passaging, but mutations and/or deletions in the eGFP gene were observed from P7, and deletions were observed from P8. It was obvious that the integrity of the recombinant viral genome maintained and virus stocks with high levels of eGFP expression could be prepared within the first six passages. These virus stocks were subsequently used in mouse infection experiments and development of high-throughput neutralization assays.

### 3.5 Characterization of the replication and pathogenicity of rSVA-eGFP in mice

**Figure 5:**
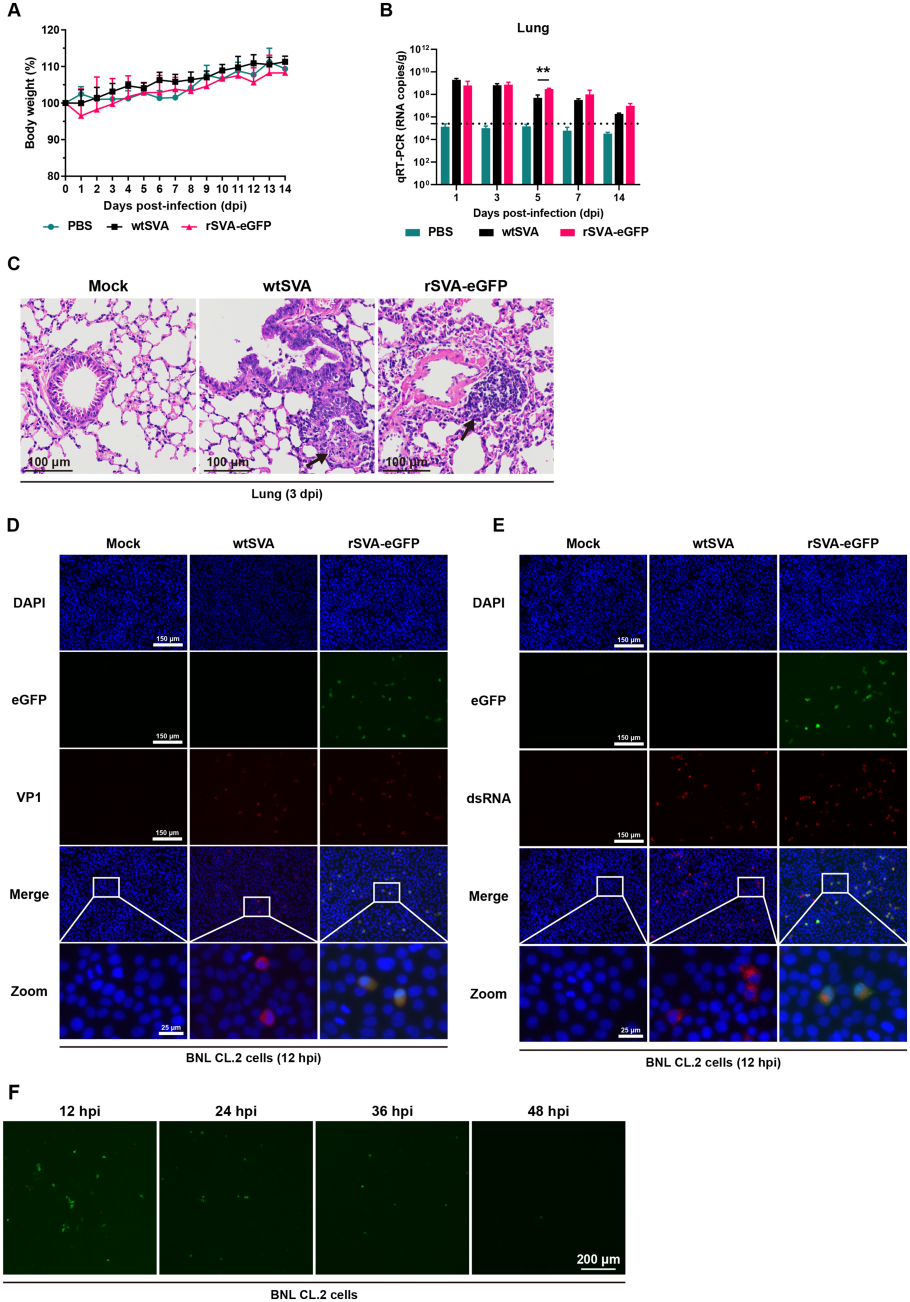
Characterization of rSVA-eGFP in mice. 3-week-old C57BL/6 J mice were mock-infected or infected with wtSVA and P5 rSVA-eGFP at 10^8^ TCID_50_, respectively. Clinical symptoms and body weight were recorded daily for 14 days, and body weight change **(A)** was generated. **(B)** The viral loads in lung tissues were measured by qRT-PCR. Dashed lines indicate the detection limit. **(C)** Histopathological analysis (hematoxylin and eosin staining) of lung tissues from mice infected with wtSVA and rSVA-eGFP at 3 dpi, as well as the control group. Arrows indicate hyperplasia and inflammatory cell infiltration in the alveoli. **(D,E)** BNL CL.2 cells were mock-treated or infected with wtSVA and P5 rSVA-eGFP (MOI = 1), respectively, and immunostained with anti-VP1 **(D)** and anti-dsRNA antibodies **(E)** at 12 hpi. Cell nuclei were stained with DAPI. **(F)** The live fluorescence-expressing BNL CL.2 cells at 12, 24, 36 and 48 hpi with P5 rSVA-eGFP (MOI = 1) were examined and presented. Statistical differences were determined using two-way analysis of variance (*p < 0.05, **p < 0.01).

To investigate whether the insertion of eGFP gene would alter SVA virulence in mice, 3-week-old C57BL/6 J mice were infected with wtSVA and P5 rSVA-eGFP at 10^8^ TCID_50_. All mice infected with wtSVA and rSVA-eGFP as well as in the PBS control group showed no weight loss, morbidity and mortality (Figure 5A). Both wtSVA and rSVA-eGFP showed a similar replication efficiency and the same tissue tropism. Viral loads were below the limit of detection in all other tissues except the lungs over the course of infection (Figure 5B; Supplementary Figure S1). The viral loads in the lungs could reach 10^9^ copies/g (Figure 5B), but there was no significant difference between wtSVA and rSVA-eGFP in most time points except at 5 dpi (*p* > 0.05) (Figure 5B). The overall lung viral loads continued to decline from 1 dpi to 14 dpi (Figure 5B). Consistently, histological analysis of all organs from mice infected with both viruses revealed lesions only in the lungs with obvious pathological changes, including hyperplasia and inflammatory cell infiltration in the alveoli (Figure 5C; Supplementary Figure S2).

Although immunofluorescence staining showed that both wtSVA and rSVA-eGFP replicated in mouse-derived BNL CL.2 cells, only a few fluorescence-positive cells were observed at 12 hpi after rSVA-eGFP infection and continued to decrease from 24 to 48 hpi (Figures 5D–F). In addition, examination of frozen sections of the lungs from rSVA-eGFP infected mice by fluorescent microscopy showed no detection of the eGFP fluorescence signal (data not shown). It is inferred that wtSVA and rSVA-eGFP were yet to acquire partial or full adaptability to mouse, so that virus replication was restricted facing clearance by the immune system, resulting in very low levels of eGFP expression. On the other hand, as rSVA-eGFP and wtSVA were shown to share the same tissue tropism and similar degrees of replication ability in mice, it demonstrated that insertion of the reporter gene did not change the original biological characteristics of the virus in mice. The construction strategy used in this study would be applicable to the construction of a reporter virus from a mouse-adapted SVA.

### 3.6 Establishment of a high-throughput neutralization assay based on rSVA-eGFP

**Figure 6:**
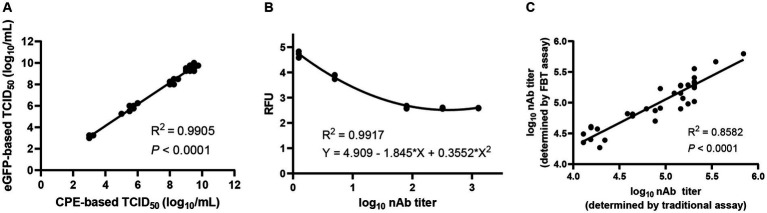
Establishment of a high-throughput neutralization assay based on rSVA-eGFP. **(A)** Correlation between CPE-based and eGFP-based TCID_50_ assays. The two assays were highly consistent (*R^2^* = 0.9905), demonstrating that eGFP signals could be used in a TCID_50_ assay to determine the rSVA-eGFP viral titers. **(B)** The standard curve of FBT assay was obtained by nonlinear fitting of RFU and nAb titer. **(C)** Correlation between the traditional eGFP-based neutralization assay and the FBT assay.

As a commonly used, accurate and reliable method for the determination of nAb titers, the traditional neutralization assay is not suitable for high-throughput titration of large numbers of clinical samples. To overcome this obstacle, an eGFP-based TCID_50_ assay using eGFP reporter signals as the readout was established by testing 17 rSVA-eGFP virus samples with both CPE-based and eGFP-based TCID_50_ assays, showing good correlation between the two assays (*R*^2^ = 0.9905) (Figure 6A; Supplementary Figure S3A). By measuring the fluorescence signals of each dilution using Varioskan LUX multimode microplate reader and developing the FBT standard curve by nonlinear fitting of RFU and nAb titers, a high-throughput FBT assay was subsequently established (Supplementary Figure S3B). High correlation (*R*^2^ = 0.9917) between the eGFP expression and the nAb titers was obtained (Figure 6B).

The feasibility of this FBT assay for high-throughput determination of nAb titers was evaluated by comparing the nAb titers of 34 unknown mouse sera using the eGFP-based neutralization assay and the FBT assay, showing good agreement between the two assays (Figure 6C; Supplementary Figure S4). The slightly higher calculated titers based on the FBT assay may reflect the higher sensitivity of the machine-based fluorescence reading. These results demonstrated that the FBT assay based on rSVA-eGFP can be used for high-throughput determination of nAb titers from a large number of serum and other clinical samples.

## 4 Discussion

SVA is an emerging pathogen that is related to vesicular diseases and has been detected in pigs and cattle. Due to its wide geographical distribution, certain host adaptability and the current lack of commercially available vaccines or drugs, SVA poses a threat to the global swine industry that cannot be ignored (Zhang et al., 2018; Liu et al., 2020b; Zhou et al., 2021). Construction of recombinant viruses expressing reporter genes will facilitate studies of viral replication, pathogenesis, and development of antiviral drugs and vaccines (Zhang H. L. et al., 2019; Wang T. et al., 2020; Chiem et al., 2022; Xing et al., 2022). In this study, we establish a stable reverse genetics system from a local isolate of SVA, and rescue a recombinant rSVA-eGFP expressing eGFP as the reporter gene. The recombinant virus shares similar biological and pathological properties with the parental virus *in vitro* cell culture and in mice. Based on this recombinant virus, a FBT assay was developed for high-throughput analysis and titration of nAb.

Various strategies have been reported for the construction of SVA infectious clone systems and rescue of rSVA expressing reporter genes. In previous studies, only the reporter gene and 2A sequence have been inserted directly between 2A and 2B genes in the viral genome (Poirier et al., 2012; Chen et al., 2016; Li et al., 2019; Liu et al., 2020a, 2021; Guo et al., 2021; Wang et al., 2024). This strategy exploits the ribosomal skipping activity of the 2A sequence that mediates the production of reporter-2A fusion protein without affecting the function of other viral proteins (Liu et al., 2017). Our attempts to rescue a recombinant virus from this isolate by direct insertion of the reporter gene and 2A sequence between 2A and 2B genes failed, supporting a few of previous reports that direct insertion of the reporter gene and the 2A sequence into the SVA genome may result in the skipping failure of 2A (Poirier et al., 2012; Wang et al., 2021). It is likely that the inserted C-terminal region of eGFP immediately upstream of 2A may reduce the P2A ribosomal skipping activity, resulting in the production of an eGFP-P2A-2B fusion protein that may affect the original function of 2B protein (De Felipe et al., 2010). To overcome the skipping inefficiency of 2A, this study used the strategy, as so far the first report, to introduce an optimal GSG linker in the construction of rSVA-eGFP. Insertion of the GSG linker would separate the eGFP protein from P2A sequence, improve the P2A skipping efficiency and eventually led to the successful rescue of the reporter virus. In addition, we also referred to strategies for the construction of recombinant EV71 and CA16 reporter viruses (Caine and Osorio, 2017; Yu et al., 2023), inserting the reporter gene and related elements between the 5′UTR and L gene, but ultimately failed to rescue a recombinant virus. By optimizing these factors, this study concludes that the genomic position between 2A and 2B genes and a GSG linker between eGFP and the P2A element were the appropriate insertion site and linker for efficient and relatively stable expression of eGFP.

Maintenance of the genetic stability of the reporter gene is a significant challenge for the construction of recombinant picornaviruses. Consistent with the previously reported eGFP-SVA (Liu et al., 2020a), the rescued rSVA-eGFP in this study exhibited eGFP deletion during serial passages, probably due to the concurrence of recombination events in the SVA genome. Genome recombination through copy-choice mechanism is one of the replication patterns of picornaviruses, whereby viral nucleotide sequences can be exchanged among different genomes (Kempf et al., 2019; Liu et al., 2022), resulting in the poor accommodation of exogenous genes by SVA and the subsequent loss of reporter genes during serial passages in cell culture. The rescued rSVA-eGFP with the optimized insertion site and GSG linker in this study can stably retain the eGFP reporter gene and maintain high-level eGFP expression within the first six passages. This relative stability makes it a useful tool for SVA virological studies.

One of the major applications of recombinant viruses expressing reporter genes in studying viral infection biology would be to trace viral replication dynamics in a mouse model. However, the limited replication of SVA in mice has impeded the application of rSVA-eGFP in such studies. As demonstrated in this report, infection with both wtSVA and rSVA-eGFP did not cause morbidity or mortality in mice. The two viruses only replicated and developed pathological changes in the lungs, demonstrating their preferential tissue tropism for this organ. Moreover, in consistency with the previously reported GFP-SVA-infected mice (Poirier et al., 2012), no eGFP fluorescence signal was observed in the lung sections from rSVA-eGFP-infected mice in this study. It confirmed that the SVA isolate used and the rescued recombinant viruses are yet to acquire mouse-adaptation, exhibiting low levels of viral replication in normal tissues. This also makes it impossible, as initially planned, to construct recombinant viruses carrying reporter genes from a mouse-adapted SVA and to establish an SVA mouse model for systematic studies of SVA replication dynamics and transmission patterns *in vivo* (Caine and Osorio, 2017). Nevertheless, as SVA is an oncolytic virus and was reported to be able to replicate in xenografts (Corbett et al., 2022; Kennedy et al., 2022), one alternative application prospect of this rSVA-eGFP would be to trace viral replication and infection dynamics in mouse xenograft models.

Another important application of rSVA-eGFP would be in high-throughput analysis of serum nAb titers. Currently, a variety of SVA serological assays have been developed, including virus neutralization test (VNT), plaque reduction neutralization test (PRNT), indirect fluorescent antibody test (IFA), fluorescent microsphere immunoassay (FMIA), indirect enzyme-linked immunosorbent assay (ELISA) and competitive ELISA (cELISA) (Dvorak et al., 2016; Joshi et al., 2016; Maggioli et al., 2018; Bai et al., 2021; Ma et al., 2023). Among them, VNT and PRNT are the gold standard for detecting and titrating nAb, but are tedious and time-consuming, making it difficult to apply these techniques in routine analysis of large serum samples. By exploiting the fluorescence expression of rSVA-eGFP as a surrogate readout for viral replication, this study has successfully established the eGFP-based neutralization assay and the FBT assay, based on rSVA-eGFP. Although the eGFP-based neutralization assay shares the advantages of accuracy and reliability with the standard neutralization assay, detection and interpretation of the fluorescence signals were time-consuming, laborious, and unsuitable for high throughput applications. Based on the standard curve and the eGFP signal intensity to calculate the nAb titers, the FBT assay enabled high-throughput detection of nAbs. It was noted that, however, the calculated nAb titers based on the FBT assay were generally slightly higher than those based on the traditional assay, probably due to the higher sensitivity of the microplate reader. More systematic and larger-scale comparison of the two assays would be required to address this issue further. To the best of our knowledge, this is the first report on the establishment of an FBT assay with rSVA-eGFP as a readout for high-throughput titration of SVA nAb.

In summary, we have rescued a recombinant SVA-eGFP virus with relatively good genetic stability and efficient expression of eGFP, based on an SVA reverse genetics system. The biological and pathological characteristics of the recombinant SVA-eGFP virus were similar to those of the parental virus *in vitro* cell culture and in mice. Furthermore, an FBT assay was developed based on rSVA-eGFP to achieve high-throughput detection of SVA nAb, demonstrating excellent concordance with the standard neutralization assay. This made the FBT assay a promising replacement for traditional neutralization assay in large-scale nAb screening, providing a highly efficient and dependable tool for SVA epidemiological research and vaccine development.

## Data Availability

The original contributions presented in the study are included in the article/Supplementary material, further inquiries can be directed to the corresponding authors.

## Supplementary material

Figure S1 The viral loads in liver, spleen, brain, heart, and kidney tissues were measured by qRT-PCR. Dashed lines indicate the detection limit.

Figure S2 Histopathological analysis (hematoxylin and eosin staining) of liver, spleen, brain, heart, and kidney tissues from mice infected with wtSVA and rSVA-eGFP at 3 dpi, as well as the control group.

Figure S3 The development of rSVA-eGFP-based neutralization assay. **(A)** Experimental scheme of CPE-based and eGFP-based TCID_50_ assays. 17 rSVA-eGFP virus samples were tested with both CPE-based and eGFP-based TCID_50_ assays to determine whether the eGFP reporter signals could replace CPE as the readout for TCID_50_. **(B)** Experimental scheme of rSVA-eGFP-based neutralization assay to measure nAb titers. BHK-21 cells were inoculated into a 96-well plate the day before the experiment (Day 0), inactivated mouse serum (at 56°C for 30 minutes) was serially diluted and mixed with 200 TCID_50_ of P5 rSVA-eGFP at 1:1, incubated at 37°C for 1 hour, and then used to infect BHK-21 cells (Day 1). The eGFP signals were examined by fluorescent microscopy at 48 hpi (Day 3) and the nAb titers were calculated by Reed-Muench method. **(C)** BHK-21 cells were infected with the neutralized mixture of rSVA-eGFP and different dilutions of serum and observed by fluorescent microscopy. The expression of eGFP gradually decreased as the antibody titers increased.

Figure S4 The nAb titers of 34 unknown mouse sera were determined by the traditional eGFP-based neutralization assay.
