# Supplementary figures and images for: Development and characterization of a recombinant Senecavirus A expressing enhanced green fluorescent protein
Source: Front Microbiol. 2024 Sep 26;15:1443696. doi: 10.3389/fmicb.2024.1443696 (PMC11464439; doi:10.3389/fmicb.2024.1443696)

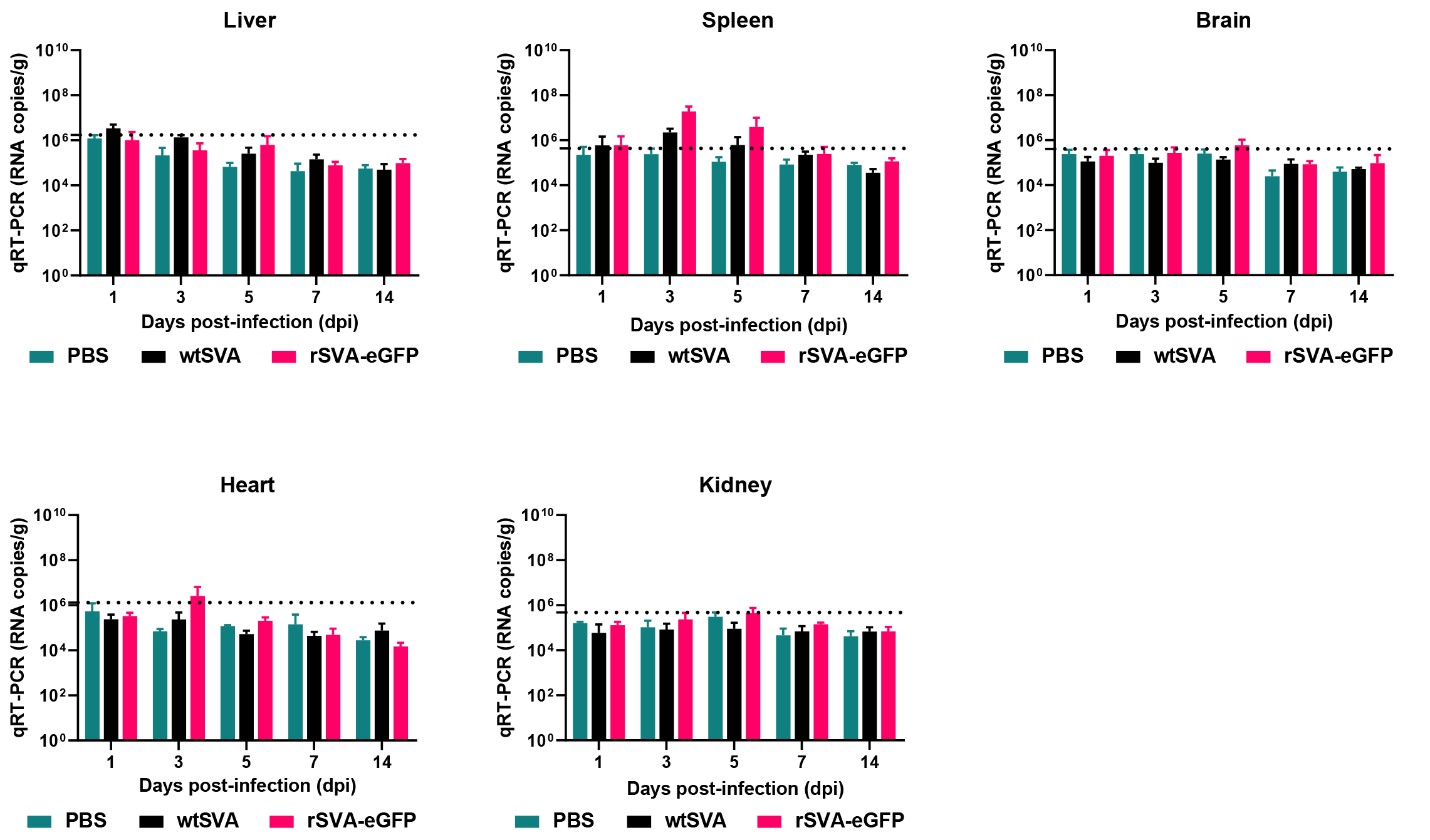

Supplement: Figure S1 — The viral loads in liver, spleen, brain, heart, and kidney tissues were measured by qRT-PCR. Dashed lines indicate the detection limit. [file Image_1.tif]

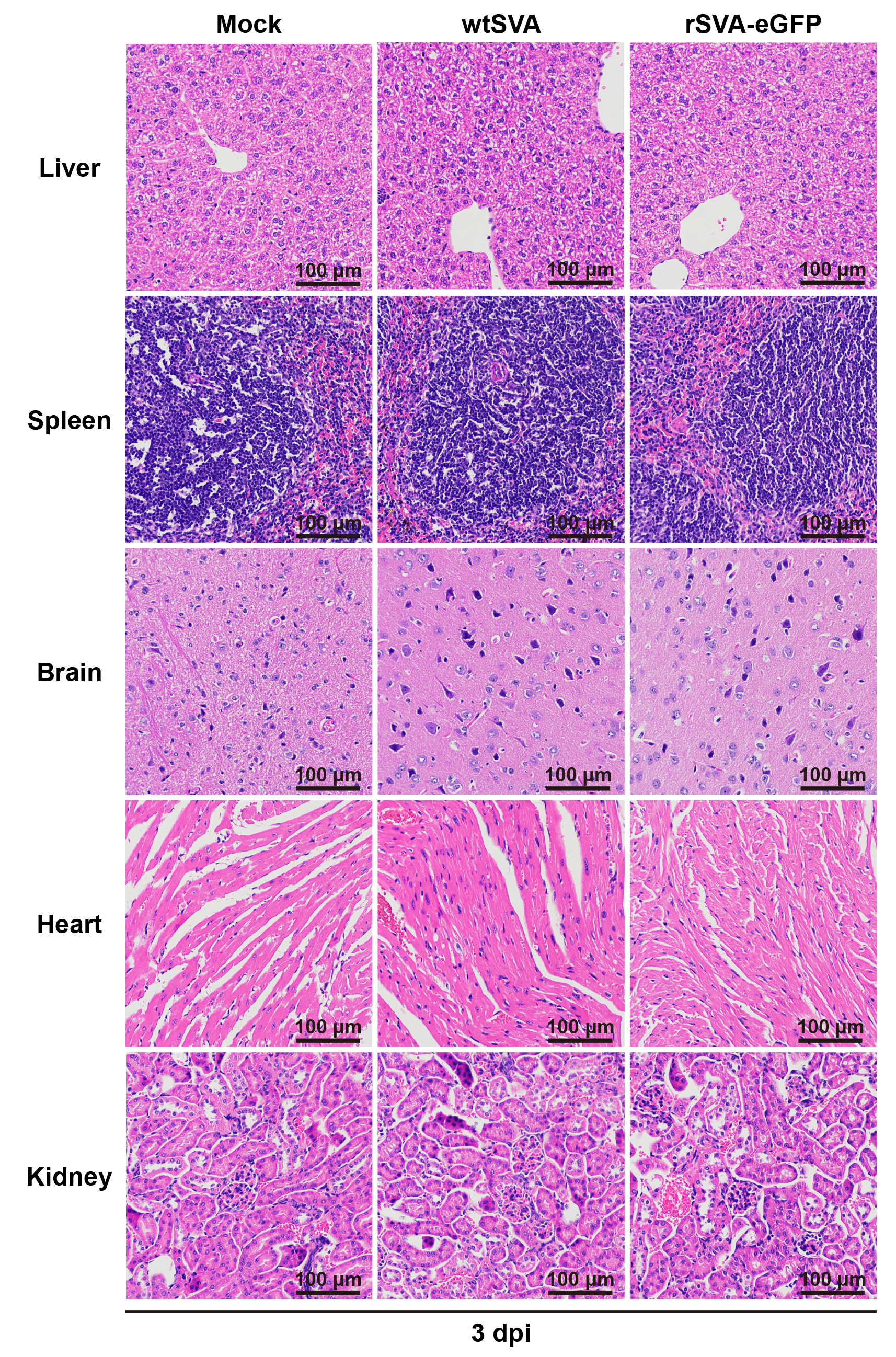

Supplement: Figure S2 — Histopathological analysis (hematoxylin and eosin staining) of liver, spleen, brain, heart, and kidney tissues from mice infected with wtSVA and rSVA-eGFP at 3 dpi, as well as the control group. [file Image_2.tif]

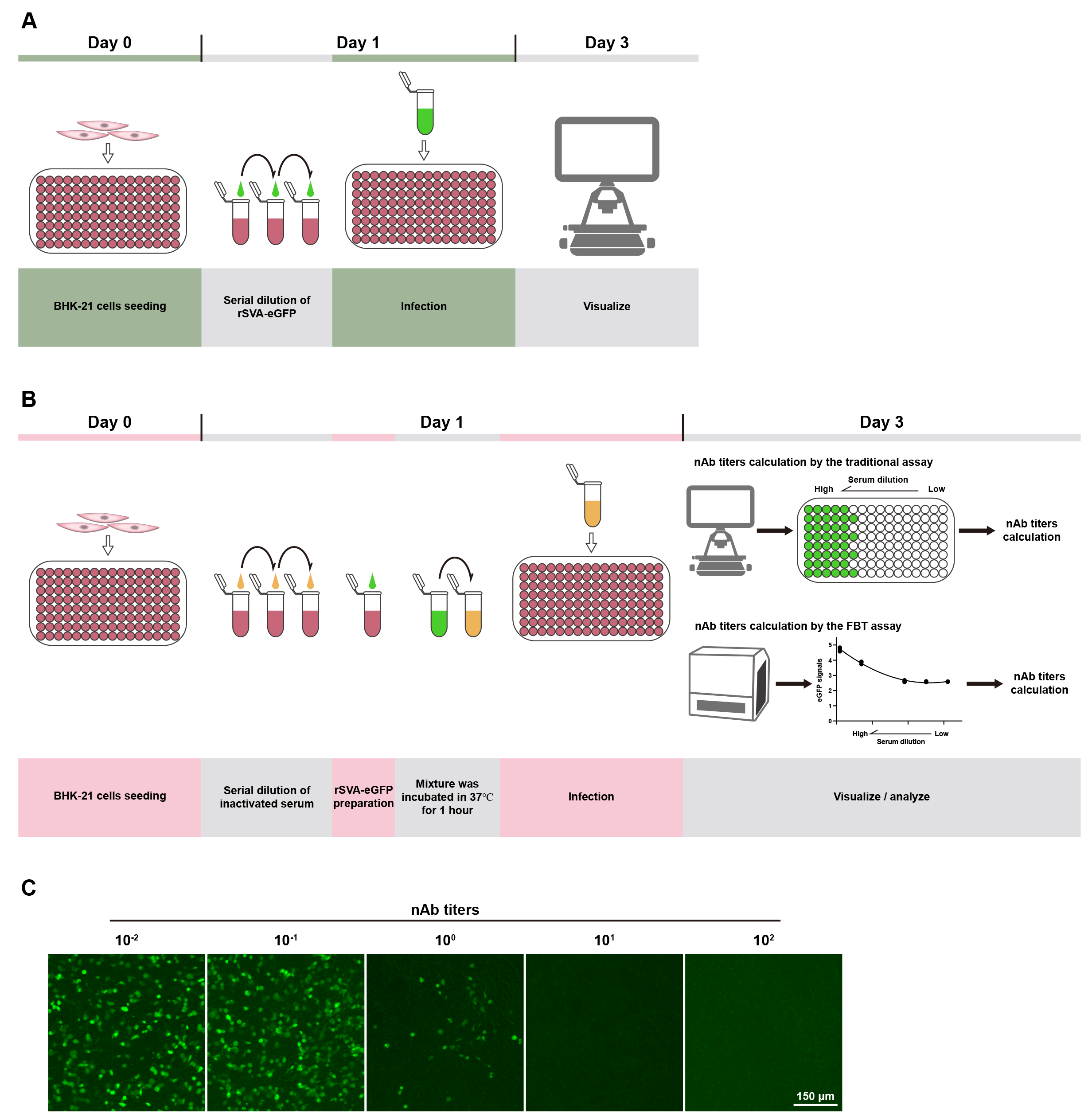

Supplement: Figure S3 — The development of rSVA-eGFP-based neutralization assay. (A) Experimental scheme of CPE-based and eGFP-based TCID50 assays. 17 rSVA-eGFP virus samples were tested with both CPE-based and eGFP-based TCID50 assays to determine whether the eGFP reporter signals could replace CPE as the readout for TCID50. (B) Experimental scheme of rSVA-eGFP-based neutralization assay to measure nAb titers. BHK-21 cells were inoculated into a 96-well plate the day before the experiment (Day 0), inactivated mouse serum (at 56°C for 30 minutes) was serially diluted and mixed with 200 TCID50 of P5 rSVA-eGFP at 1:1, incubated at 37°C for 1 hour, and then used to infect BHK-21 cells (Day 1). The eGFP signals were examined by fluorescent microscopy at 48 hpi (Day 3) and the nAb titers were calculated by Reed-Muench method. (C) BHK-21 cells were infected with the neutralized mixture of rSVA-eGFP and different dilutions of serum and observed by fluorescent microscopy. The expression of eGFP gradually decreased as the antibody titers increased. [file Image_3.tif]

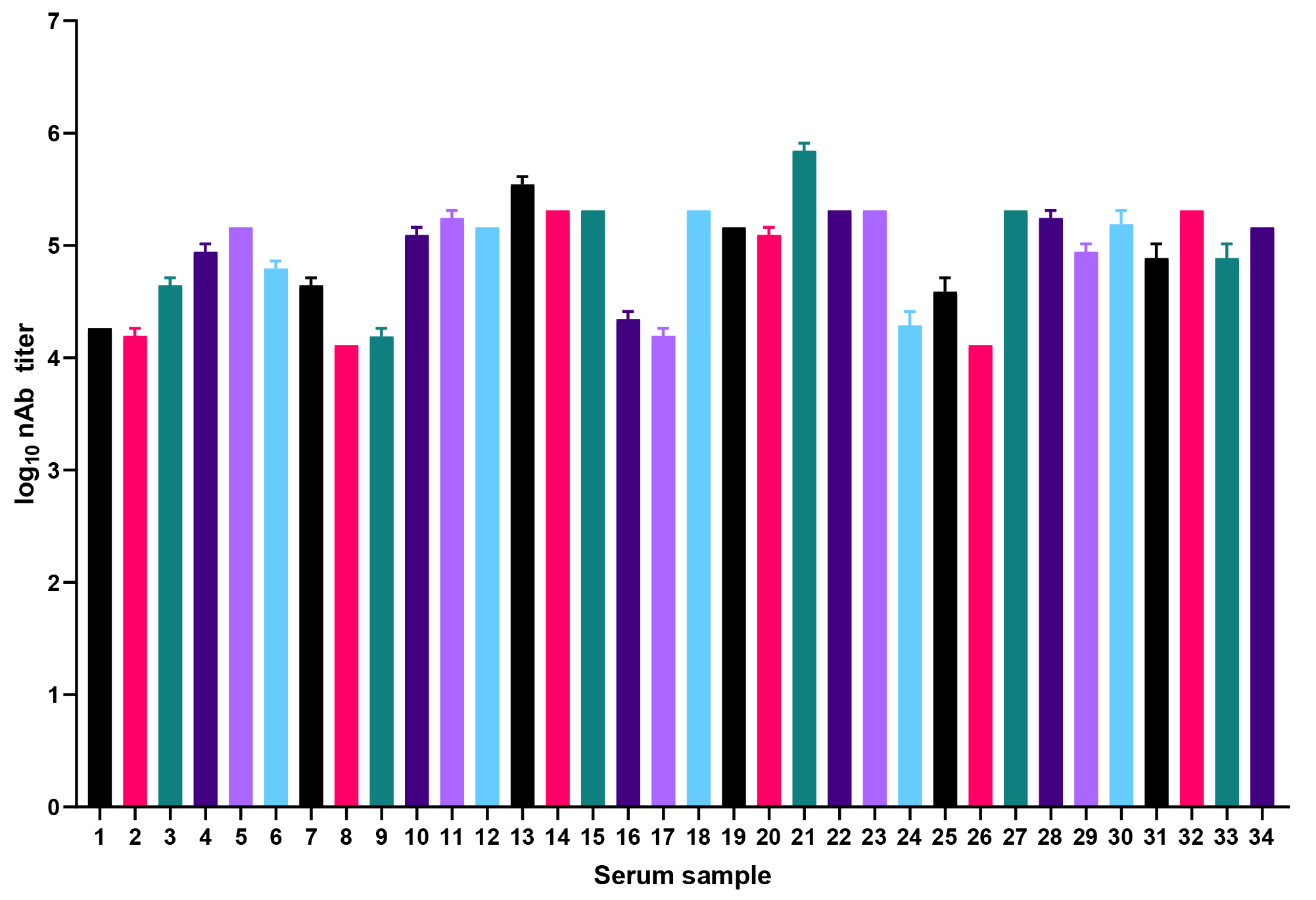

Supplement: Figure S4 — The nAb titers of 34 unknown mouse sera were determined by the traditional eGFP-based neutralization assay. [file Image_4.tif]
