# Supplementary material for: Development and characterization of a recombinant Senecavirus A expressing enhanced green fluorescent protein
Source: Front Microbiol. 2024 Sep 26;15:1443696. doi: 10.3389/fmicb.2024.1443696 (PMC11464439; doi:10.3389/fmicb.2024.1443696)
Supplement: Supplementary file 5 [file Table_1.DOCX]

Supplementary Material

**Supplementary Table 1**

Primers used in the study.

| Primers | Primer sequence (5′-3′) |
| --- | --- |
| Fragment A-F | gactcaagcgcggaaagcgctGTAACCACATGCTGTTAGTCCCTTT |
| Fragment A-R | ggaagagtggcgaagttcgaaCTGATGGCGCCTACCAGGG |
| Fragment B-F | cttcgccactcttcctccggaCCCAGACTTCTTCGACGGATAC |
| Fragment B-R | gtcaccgtaggcgatgatatcAACCATGTCATATTCAAATTCCTTGT |
| Fragment C-F | ggtaggcgccatcagttcgaaCTTCGCCAACTACCGGGG |
| Fragment C-R | tcgaagaagtctgggtccggaGGAAGAGAATACACACTTTGTGAGCC |
| eGFP-F | GTGGCGTTCTCGCTAATAC |
| eGFP-R | GGAGGCGGTTCTACAGTAAAG |
| qPCR-F | GGCACTGGCTCCTTCGAGG |
| qPCR-R | GGTCGCGGCACAACCAG |
