# Supplementary material for: Development and characterization of a recombinant Senecavirus A expressing enhanced green fluorescent protein
Source: Front Microbiol. 2024 Sep 26;15:1443696. doi: 10.3389/fmicb.2024.1443696 (PMC11464439; doi:10.3389/fmicb.2024.1443696)
Supplement: Supplementary file 6 [file Table_2.docx]

Supplementary Material

**Supplementary Table 2**

Elements used in the construction of pSVA-eGFP.

| Elements | Sequence (5′-3′) |
| --- | --- |
| porcine teschovirus-1 2A (P2A) | GCTACCAACTTCAGCCTGCTGAAGCAGGCCGGAGACGTGGAGGAGAACCCTGGCCCT |
| GSG linker | GGCTCTGGA |
| SSG linker | AGCTCTGGA |
| GGG linker | GGCGGTGGA |
