# Supplementary material for: Development and characterization of a recombinant Senecavirus A expressing enhanced green fluorescent protein
Source: Front Microbiol. 2024 Sep 26;15:1443696. doi: 10.3389/fmicb.2024.1443696 (PMC11464439; doi:10.3389/fmicb.2024.1443696)
Supplement: Supplementary file 7 [file Table_3.docx]

Supplementary Material

**Supplementary Table 3**

Information about inserted fragments in infectious clones.

| Infectious clones | Length of parental SVA genome (bp) | Composition of inserted fragments | Length of Inserted fragments (bp) | Ratio of inserted fragments to SVA genome (%) |
| --- | --- | --- | --- | --- |
| pSVA-eGFP-1 | 7319 | eGFP gene and P2A | 774 | 10.58 |
| pSVA-eGFP-2 |  | eGFP gene, GSG linker and P2A | 783 | 10.70 |
| pSVA-eGFP-3 |  | eGFP gene, SSG linker and P2A | 783 | 10.70 |
| pSVA-eGFP-4 |  | eGFP gene, GGG linker and P2A | 783 | 10.70 |
| pSVA-eGFP-5 |  | eGFP gene, GSG linker and P2A | 783 | 10.70 |
